# The HuMet Repository: Watching human metabolism at work

**DOI:** 10.1016/j.celrep.2024.114416

**Published:** 2024-07-20

**Authors:** Patrick Weinisch, Johannes Raffler, Werner Römisch-Margl, Matthias Arnold, Robert P. Mohney, Manuela J. Rist, Cornelia Prehn, Thomas Skurk, Hans Hauner, Hannelore Daniel, Karsten Suhre, Gabi Kastenmüller

**Affiliations:** 1 Institute of Computational Biology, Helmholtz Zentrum München - German Research Center for Environmental Health, Neuherberg, Germany; 2 Digital Medicine, University Hospital of Augsburg, Augsburg, Germany; 3 Department of Psychiatry and Behavioral Sciences, Duke University, Durham, NC, USA; 4 Metabolon, Inc., Durham, NC, USA; 5 Department of Physiology and Biochemistry of Nutrition, Max Rubner-Institut, Karlsruhe, Germany; 6 Metabolomics and Proteomics Core, Helmholtz Zentrum München - German Research Center for Environmental Health, Neuherberg, Germany; 7 ZIEL Institute for Food and Health, Core Facility Human Studies, Technical University of Munich, Freising, Germany; 8 Else Kröner Fresenius Center for Nutritional Medicine, School of Life Sciences, Technical University of Munich, Freising, Germany; 9 Institute for Nutritional Medicine, School of Medicine and Health, Technical University of Munich, Munich, Germany; 10 School of Life Sciences, Technical University of Munich, Freising, Germany; 11 Department of Biophysics and Physiology, Weill Cornell Medicine - Qatar, Doha, Qatar; 12 Lead contact

## Abstract

Metabolism oscillates between catabolic and anabolic states depending on food intake, exercise, or stresses that change a multitude of metabolic pathways simultaneously. We present the HuMet Repository for exploring dynamic metabolic responses to oral glucose/lipid loads, mixed meals, 36-h fasting, exercise, and cold stress in healthy subjects. Metabolomics data from blood, urine, and breath of 15 young, healthy men at up to 56 time points are integrated and embedded within an interactive web application, enabling researchers with and without computational expertise to search, visualize, analyze, and contextualize the dynamic metabolite profiles of 2,656 metabolites acquired on multiple platforms. With examples, we demonstrate the utility of the resource for research into the dynamics of human metabolism, highlighting differences and similarities in systemic metabolic responses across challenges and the complementarity of metabolomics platforms. The repository, providing a reference for healthy metabolite changes to six standardized physiological challenges, is freely accessible through a web portal.

## In brief

Weinisch et al. introduce the HuMet Repository (https://humet.org), a web-based resource for exploring dynamic metabolic responses of healthy subjects to six physiological stimuli, including prolonged fasting, oral glucose/lipid loads, and exercise. The repository enables searching, analyzing, and visualizing time-resolved metabolomics data comprising 2,656 metabolites in human blood, urine, or breath.

## Graphical Abstract

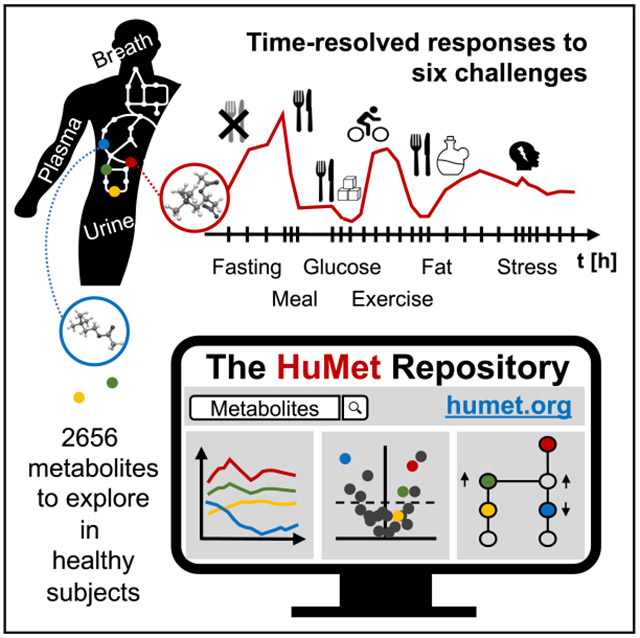

## INTRODUCTION

The human body continually adapts and dynamically responds to physiological perturbations and challenges, such as dietary intake, physical activity, or stress.^1^ Impaired metabolic flexibility is a hallmark of many metabolic disorders, including type 2 diabetes and cardiovascular diseases.^2^ It leads to aberrations from the “normal,” healthy response to challenges in patients. To differentiate between such atypical metabolic responses and the normal adaptation of metabolism in a healthy state, detailed knowledge of metabolism’s typical, healthy dynamics and its variance across individuals is crucial.

Metabolomic profiles in accessible body fluids such as blood or urine provide snapshots of a person’s metabolic state at a given time.^3^ Multiple snapshots taken at time points (TPs) during or immediately after a specific challenge (e.g., extended fasting, exercise, or eating a fat- or carbohydrate-rich meals) allows time-resolved monitoring of the systemic metabolic adaptation, i.e., metabolomics enables us to watch metabolism “at work” in healthy and patient cohorts.

However, most metabolomics studies involving challenges collect only two samples, before and after (or under) the challenge. Only a few studies have described the normal dynamic responses to standardized or all-day metabolic stressors, including different nutritional challenges^4-7^ or exercise,^7-9^ in detail using time-resolved metabolomics. The HuMet study^7^ was specifically designed to capture the dynamics of metabolism in a homogeneous, healthy group (*n* = 15) across multiple challenges, including four highly standardized (i.e., reproducible) nutritional challenges, physical exercise, and a stress test. Due to their high dimensionality, accessing and leveraging the time-resolved metabolomics data from these studies remains challenging even for simple research questions, particularly for researchers who are not used to handling big and complex longitudinal datasets.

**Figure 1.**
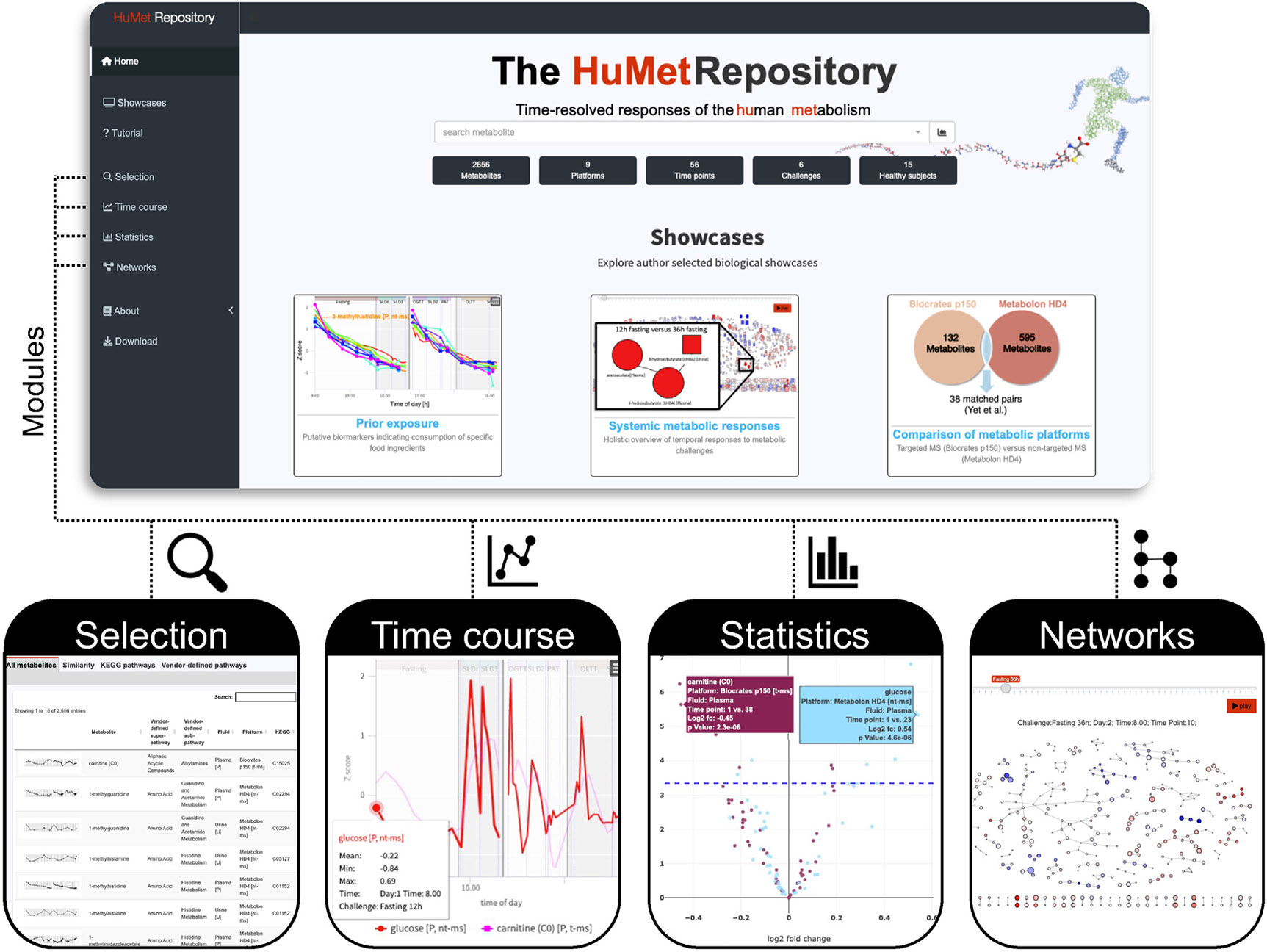
HuMet Repository frontend The HuMet Repository (https://humet.org) integrates four modules to explore the time-resolved metabolomics data of the HuMet study, reflecting responses to physiological challenges in healthy individuals. In the selection module, the user can select metabolites from a table, with options for sorting and filtering by metabolite properties, including time course similarities. Line plots within the time course module visualize time-resolved metabolite profiles of participants, providing multiple options for data transformation and representation. Plots within the statistics module depict statistical results from multiple analyses. The networks module offers a holistic overview of metabolite changes within predefined and reconstructed biological pathways.

We here describe the HuMet Repository, a public online resource that allows intuitive, interactive exploration and visualization of a comprehensive time-resolved metabolomics dataset capturing the normal dynamics of metabolism in men (Figure 1). To build this resource, we profiled samples from the HuMet study cohort^7^ using five complementary non-targeted mass spectrometry-based (nt-MS) metabolomics and lipidomics methods. These analyses resulted in time-resolved response data for 2,179 analytes. Combined with the pre-existing data for 477 metabolites, the HuMet Repository contains temporal profiles for, in total, 2,656 metabolites measured in blood, urine, or breath samples from 15 healthy, young males who engaged in the 4-day HuMet trial with six different metabolic challenges and samples collected at up to 56 TPs for each participant. For each challenge, we identify metabolites and groups of metabolites that change using univariate statistics as well as data-derived metabolic networks. A dedicated web-based interface enables users to search, browse, and visualize this complex dataset for further explorative analysis without specific computational expertise. Single *ad hoc* questions such as “Do blood levels of a metabolite of interest (e.g., a potential biomarker) change postprandially or in response to exercise? If so, when are its levels back to baseline in healthy individuals?” can thus be answered directly based on these data without any additional effort on data preprocessing or analysis. In three showcases, we exemplify the use of the HuMet Repository: (1) applying the implemented search functions, we identify metabolites with similar trajectories, e.g., metabolites that show a steady decrease over the study phase and presumably stem from exposure before the study. (2) We check the similarity of trajectories of the same metabolites determined on two different metabolomics platforms, providing insights into the concordance of these measurements. (3) Making use of data-derived metabolic networks, we inspect the dynamic changes during extended fasting across the whole metabolism.

In summary, the interactive HuMet Repository represents a resource of time-resolved metabolomics data in response to different physiological challenges within the same healthy homogeneous population while covering a wide variety of metabolomics approaches. The HuMet Repository is freely accessible at https://humet.org/.

## RESULTS

### Deep metabotyping provides time-resolved profiles for 2,179 metabolites

To build the HuMet Repository as a comprehensive resource of time-resolved metabolomic profiles in healthy individuals, we examined 840 (15 subjects × 56 TPs) plasma and 240 (15 subjects × 16 TPs) urine samples of the HuMet study cohort^7^ using four nt-MS analytical methods (Metabolon HD4 platform^10^). This resulted in time-resolved relative quantifications for 595 and 619 metabolites in plasma and urine, respectively (Table 1), including 397 metabolites for which the time courses are available in both fluids. These metabolites span eight different metabolite classes called “super-pathways” (amino acids, carbohydrates, cofactors and vitamins, energy, lipids, nucleotides, peptides, xenobiotics) and more than 83 different metabolic pathways (“sub-pathways”). In addition, the samples of four participants were analyzed on the Lipidyzer platform, yielding quantifications of 965 molecules that provide structurally detailed information on complex lipids (see STAR Methods).

In the repository, we also included the previously published data from the initial metabolomics analysis of the HuMet samples,^7^ which covered mainly amino acids, lipids (acylcarnitines, glycerophospholipids, sphingolipids), and lipoproteins. The plasma concentrations of these metabolites were measured using the commercially available Biocrates p150 kit for targeted MS-based (t-MS) analysis. Levels of plasma lipoproteins were assessed at numares AG (formerly LipoFIT, Regensburg, Germany) applying a nuclear magnetic resonance (NMR)-based approach. Moreover, breath air and breath condensate samples had been analyzed on in-house platforms of partners from academia. This first wave of measurements resulted in quantifications for 477 metabolomic measures for HuMet samples (Table 1).

While several of the previously profiled metabolites overlap with metabolites measured as part of the newly added data (e.g., amino acids, acylcarnitines), information from the previous and new platforms is largely complementary in (1) coverage of the metabolome (with many metabolite classes added through the new data, e.g., nucleotides, carbohydrates, steroids, xenobiotics; Table 1), (2) type of quantification (with Biocrates p150 and NMR-based platforms providing absolute quantification), and (3) isobaric/isomeric resolution of lipids (e.g., measurement of isobars as sums in Biocrates p150 versus fatty acid resolution in Lipidyzer). For those metabolites measured on multiple platforms, we kept the recorded time course from each platform to allow cross-platform comparisons (see also showcase platform comparison). Descriptions of all (newly and previously measured) metabolites are provided in Table S1. Thus, in total, the HuMet Repository provides access to time-resolved data for 2,656 metabolites in plasma, urine, and breath, resulting in a total of 1.1 million data points.

### Metabolic responses to six physiological challenges

**Figure 2.**
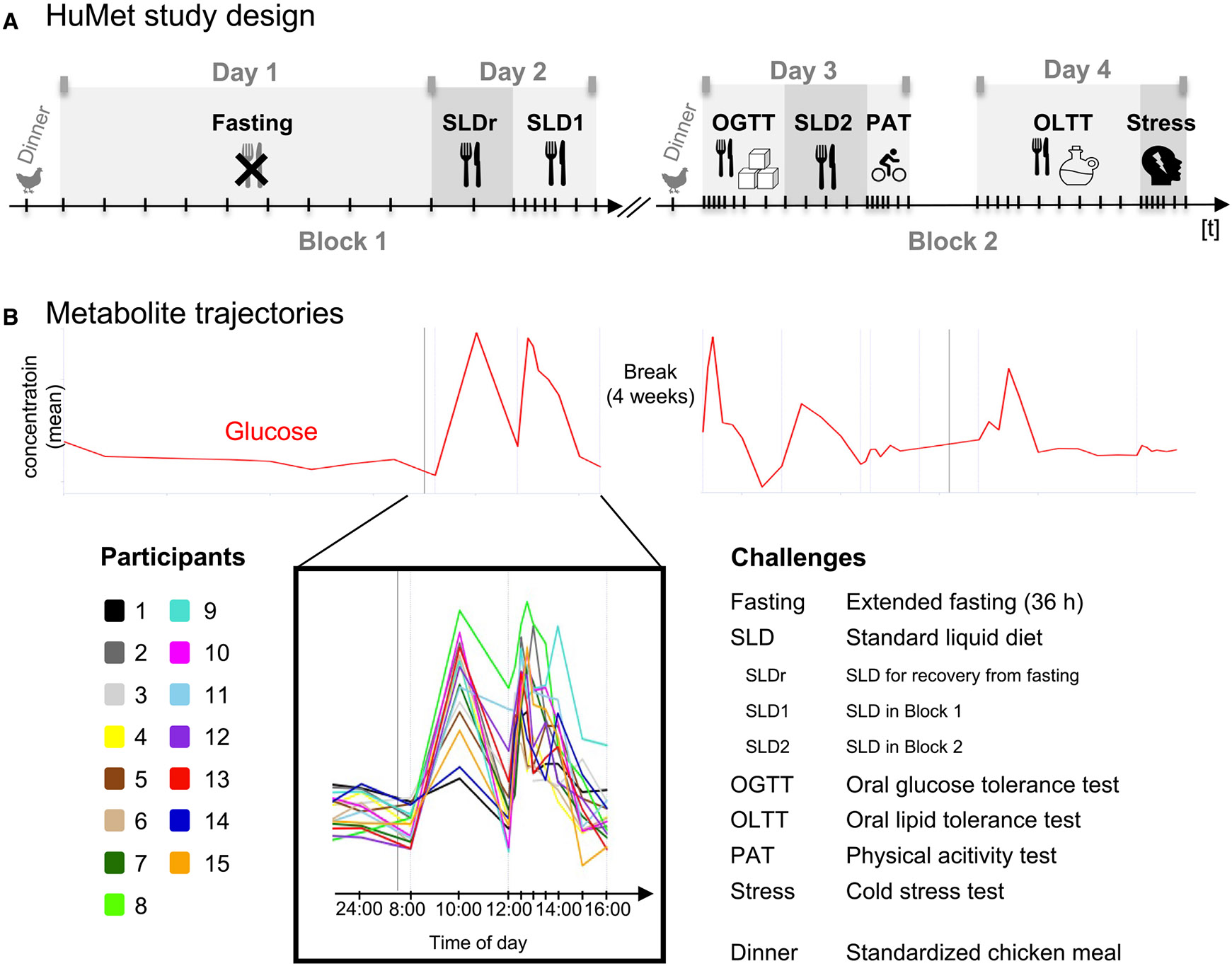
Metabolite profiles across six physiological challenges (A) A sequence of metabolic challenges was applied over two study blocks, each covering a period of 2 days. All participants had the same chicken meal for dinner at 7 p.m. on the evening before each block. Five of the six challenge tests were applied once, while participants were exposed to the mixed meal challenge three times (SLDr, SLD1, SLD2). Challenge tests along with their abbreviations as used in the scheme are listed in (B). Plasma, urine, and breath samples were collected at up to 56 time points in variable time intervals (15 min–2 h) depending on the challenge. (B) In the repository, various representations of metabolite time courses can be visualized. In the provided example, the red line represents the mean levels of plasma glucose over time, i.e., levels for the 15 study participants have been averaged at each of the 56 time points. The inset zooms into the 15 individual metabolite time courses for challenges SLDr and SLD1 (see legend), colored by study participants.

To characterize the normal, healthy dynamics in metabolism under physiological challenges, we analyzed the concentration changes of each metabolite during/after each of the six physiological challenges, which the HuMet study participants were exposed to during the 4 days of sample collection (Figure 2): in the first block of 2 days, participants fasted for 36 h (Fasting) and were allowed to recover from fasting after breakfast and a lunch consisting of a standardized drink that represents a mixed meal (SLDr, SLD1). The second block of the study, which was conducted after a 4-week break, included a physical activity test (PAT), a stress test (Stress), and three different nutritional challenges, namely an oral glucose tolerance test (OGTT) resembling a diet rich in carbohydrates, an oral lipid tolerance test (OLTT) resembling a high-fat diet, and a mixed meal (SLD2; same liquid diet as SLDr). Throughout the experiment, three different sample types (plasma, urine, and breath) were collected at up to 56 TPs in variable time intervals (15 min–2 h, depending on the challenge), enabling temporal profiling of metabolite changes during or after the six challenges for each participant (for details, see STAR Methods and Krug et al.^7^).

To identify metabolites whose abundances significantly changed in response to a challenge, we performed paired t tests for each metabolite and TP during/after the challenge (time frames are given in Table 2) compared to the challenge-specific baseline. After adjustment for multiple testing, this analysis yielded 620, 27, 117, 101, and 21 significant hits, comprising 220, 15, 66, 64, and 16 metabolites that changed at various TPs during/after extended fasting, glucose/mixed/high-fat meals, and physical activity, respectively (Table S2). Stress did not show any significant hit after correction for multiple testing (Bonferroni). Since only subtle changes, such as an increase in cortisol,^13^ are expected in response to a cold stress test, we assumed that this negative finding might be due to the limited sample size. Based on the effect estimate for cortisol, we performed a power calculation and found that 58 participants would be needed to detect this effect with 80% power when applying the Bonferroni-corrected significance threshold.

For each of the challenges, Table 2 lists those significantly altered metabolites that showed the lowest *p* value and/or largest fold change (decrease/increase) observed. As an example, the ketone body 3-hydroxybutyrate (BHBA) in urine showed the largest increase after 36-h fasting when compared to overnight (12-h) fasting (log2 fold change [log2fc] = 7.7). Significant increases of BHBA in plasma were observed after prolonged fasting before those in urine (log2fc = 3.0 after 22 h fasting), indicating the generation of ketone bodies for energy supply in this phase. Comparing the observed levels of plasma BHBA during extended fasting to those measured after the OLTT, we found similar levels of this ketone body 6 h after ingestion of the lipid-rich challenge drink. All statistical results are provided in tabular form as well as in interactive volcano plots within the HuMet Repository (statistics module).

### Data-derived metabolic networks provide molecular context for metabolite changes

To allow inspection of dynamic metabolic changes in the context of metabolic pathways and overall metabolism, we generated different types of metabolic networks covering the metabolites of the t-MS- and nt-MS-based platforms in plasma and urine (Biocrates p150, Metabolon HD4), which were applied to samples from all participants and TPs: (1) knowledge-based networks, which connect metabolites by their pathway membership based on existing pathway or metabolite class definitions (e.g., KEGG) as annotated by the providers of the metabolomics data, and (2) data-derived networks, where we built on our previous findings that connecting metabolites based on their significant partial correlations in blood and urine reconstructs known metabolic networks from cross-sectional data, yielding so-called Gaussian graphical models (GGMs).^14-16^ Applying a method that takes the longitudinal design of data into account,^17^ we constructed GGMs based on the HuMet data from blood and urine for each fluid and platform separately (single-fluid networks). For plasma, we additionally generated GGMs combining data from multiple platforms (Metabolon HD4, Biocrates p150, in-house biochemistry). For data from the Metabolon HD4 platform, we connected the plasma- and urine-specific GGMs into multi-fluid networks by linking the two nodes representing the same metabolite in each fluid by an additional edge. For example, the plasma network comprising metabolites from the Metabolon HD4 platform contains 339 edges connecting metabolites with partial correlations ≥0.12 (see STAR Methods for details on cutoffs). Merging the plasma network with the network inferred from urine metabolites (Metabolon HD4 platform, partial correlations ≥ 0.09; see STAR Methods), which consists of 227 edges, results in a multi-fluid network, where 333 edges connect the same metabolites measured in plasma and urine.

To test whether the GGMs inferred from longitudinal metabolomics data reconstruct metabolic networks similarly to GGMs inferred from cross-sectional data,^14^ we examined how close metabolites connected via an edge in the GGM were within the human biochemical reaction network. For each pair of connected metabolites of the plasma GGM, we assessed their pathway distance in KEGG,^18^ i.e., we counted the reaction steps that are needed to convert the two metabolites into each other based on known (intracellular) metabolic pathways. This analysis was possible for 74 out of the 339 edges for which both metabolites were mappable to KEGG (*n* = 129) and which were represented in a KEGG pathway of human metabolism. For 29 out of the 74 edges, the connected metabolites were also directly linked in KEGG (i.e., showed a pathway distance of 1); for 22 edges, we observed pathway distances of 2 or 3. A summary of results is provided in Table S3. In a bootstrapping approach, in which we generated 1,000 networks with the same topology but randomized node labeling, a maximum of 6 edges with pathway distance 1 was found in only one of the 1,000 networks; we obtained similar results when performing the analogous analysis of the networks reconstructed from urine metabolites (Table S4), confirming that GGMs based on the longitudinal HuMet data resemble known metabolic networks.

Using these data-derived metabolic networks, we mapped temporal changes in the abundances of metabolites by coloring nodes according to the metabolites’ log2fcs during each challenge. This mapping allows for a holistic, metabolism-wide overview of time-resolved challenge responses (see also showcase systemic metabolic responses).

### A web-based resource for data visualization and exploration

To facilitate access to data and results from the HuMet study, we set up the HuMet Repository as a web-based framework holding the complete HuMet dataset and providing four modules (selection, time course, statistics, and network) for interactive data exploration (Figure 1). The user can filter the dataset to form subsets for visualizations and analyses across these modules. Filtering includes restrictions on specific TPs, challenges, subjects, metabolomics platforms, and sample types. The user can also choose between various data transformations, including data scaling, imputation, and data representation as log2fcs. Plots generated as part of the different modules are interactive and can be downloaded along with the underlying data. Furthermore, the framework allows users to download the complete data as well as selected subsets and transformations thereof for further analysis.

The selection module allows users to select from 2,656 metabolites in the HuMet dataset with sorting and filtering options by assigned properties, including metabolite classes, metabolomics platform, biofluid, and various metabolite identifiers (ChEBI,^19^ KEGG,^18^ HMDB^20^) and synonyms. Moreover, metabolites can be selected by specifying pathways of interest using pathways as defined by either KEGG or the metabolomics platform (“annotated”). In addition, the user can search for metabolites that show a similar temporal profile to a specified reference metabolite (see also showcase prior exposure and showcase platform comparison). Thereby, metabolites can be ranked by their similarity to the reference using different distance measures (Euclidean, Manhattan, Fréchet) or correlation (Pearson). To facilitate the comparison of different sets of metabolites, the user can assign metabolites to different groups (“bags”) and toggle between them when using other modules.

In the time course module, users can visualize and compare temporal trajectories of selected metabolites over the 4 days of the HuMet study. For each metabolite, the subject-specific temporal profiles are displayed in different colors, using the same color coding for participants consistently throughout the repository (Figure 2B). The visualization is adaptive to various data transformations such as *Z* scoring, imputation of missing values, and log2fcs related to challenge baseline. Mean time courses connecting the mean levels of participants at each TP can be displayed for multiple metabolites in one plot to facilitate visual exploration and comparison of temporal changes between metabolites.

For identifying metabolites whose abundances significantly changed in response to a challenge or between two user-defined TPs, the HuMet Repository provides hypothesis testing within the statistics module. For amenable exploration, all statistical results are provided in tabular form as well as in interactive volcano plots.

The networks module allows users to inspect metabolites of interest in the context of metabolic pathways and reconstructed metabolic networks. To this end, we provide knowledge-based and data-derived networks for the metabolites from the two MS-based metabolomics platforms. For network generation based on the precalculated pairwise partial correlations between metabolites, the user can choose from different cutoffs, above which edges are drawn. For example, fixed partial correlation values can be chosen as thresholds.^21^ Alternatively, edges can be filtered based on the statistical significance of the partial correlation between the two metabolites, applying different methods to adjust for multiple testing of edges (false discovery rate, Bonferroni). These choices lead to various alternatives of single-fluid networks for plasma and urine. To allow exploring the metabolic response to the different challenges within these networks, the user can map the log2fcs of metabolites (in relation to their challenge baselines) onto the metabolite nodes in the network using red (increase) and blue (decrease) color gradients. For the generation of the multi-fluid networks based on the Metabolon HD4 plasma and urine datasets, we merged the corresponding single-fluid networks by connecting the same metabolites measured in plasma and urine by an additional edge. Using this approach, changes in urine and plasma metabolites can be displayed in parallel.

### Showcase prior exposure: Identify metabolites with washout-like temporal profiles

In the first showcase, we seek to identify metabolites that originate from exposure such as to foods or drugs, to which participants had no access during the 2 × 2 days of the study, using the HuMet Repository. Prior to each of the two blocks in the HuMet study (Figure 2), all participants ate the same “chicken with vegetables” meal (prepared from a packaged frozen instant meal) containing a complex mixture of dietary ingredients that were not included in any of the liquid meals provided during the 4-day study phase (e.g., meat or vegetables).

**Figure 3.**
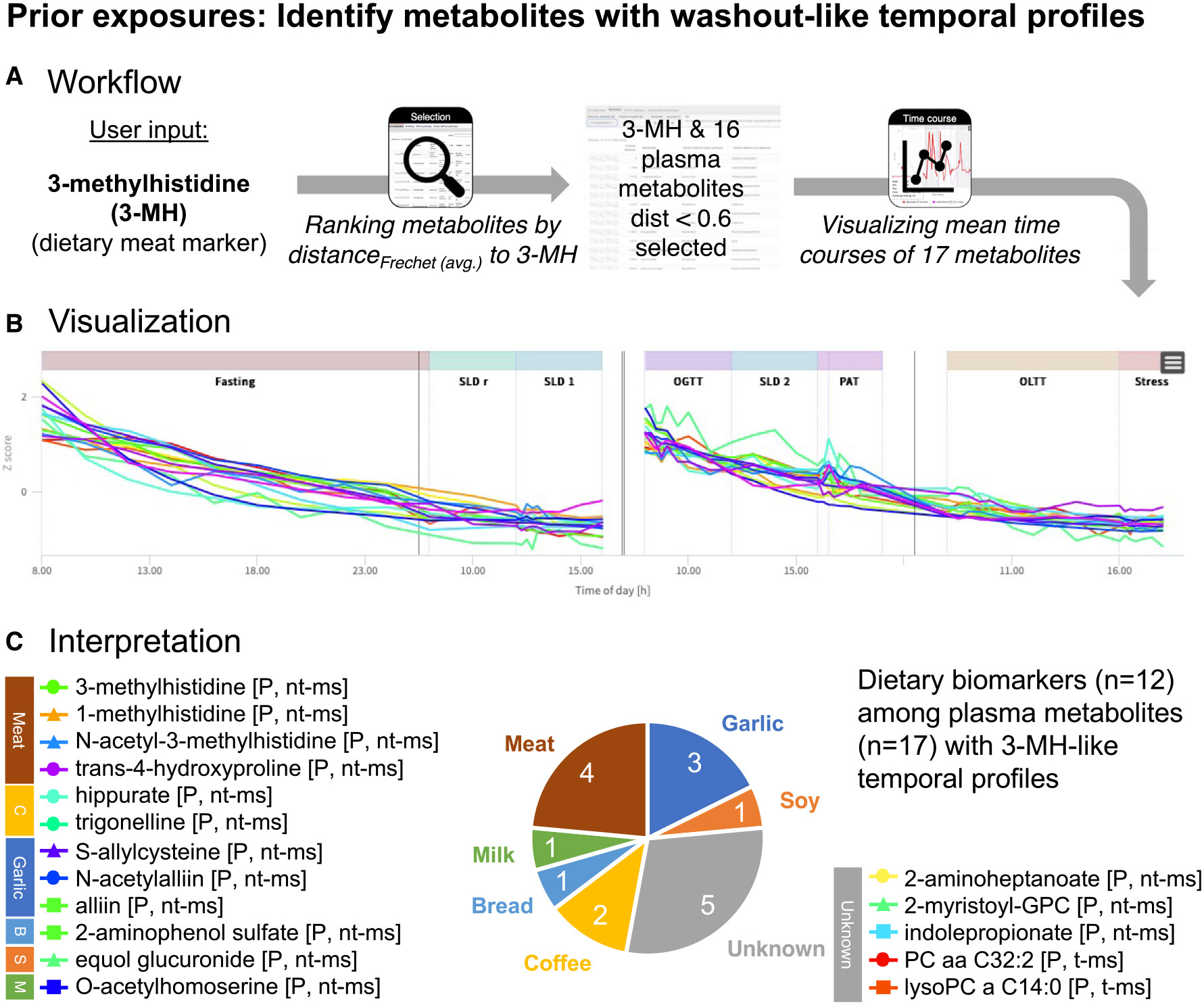
Exploration of metabolites from prior exposure (A) Workflow to identify metabolites (Metabolon HD4, Biocrates p150) with similar trajectories as the reference plasma metabolite 3-methylhistidine (3-MH), a dietary marker for meat intake, using the similarity search implemented in the selection module. (B) Time courses of 3-MH and the 16 plasma metabolites with most similar trajectories (Fréchet distance <0.6) as visualized within the time course module. (C) Out of the 17 metabolites, 12 are known biomarkers for various food items that have not been provided to participants during the study blocks, indicating exposure prior to the study.

Methylhistamines have been suggested as biomarkers that reflect chicken meat intake.^22,23^ In our participants, plasma levels of 3-methylhistidine exhibited a washout-like temporal profile with a steady decrease after chicken intake. The profiles showed minimal interference with stimuli during the study phase, which is a prerequisite for a true dietary biomarker (Figure 3). We therefore chose this metabolite as a starting point for the search of metabolites with similar kinetic characteristics, potentially indicating further prior exposure of the participants. We used the similarity search option in the selection module to rank metabolites (Metabolon HD4, Biocrates p150) by the distances of their temporal profiles to the reference profile of 3-methylhistidine in plasma.

We found S-allylcysteine in plasma to be the metabolite with the most similar temporal profile, showing a distance (Fréchet) of 0.2712 from plasma 3-methylhistidine; 34 additional metabolites had distances less than 0.6, with 16 being plasma metabolites. Out of these 16 plasma metabolites, 12 are metabolites (or direct derivatives of metabolites) listed in FooDB^24^ and/or are linked to food-related exposure in the Exposome-Explorer.^25^ These 12 metabolites indicate putative exposure to meat, garlic, bread, coffee, milk, and soy (Table S5). Most of these metabolites were detectable in almost all participants and at most TPs. In contrast, equol glucuronide was only detected in two individuals (subjects 1 [6 TPs of the first block] and 8 [all TPs]), respectively. Equol is generated from daidzein, an isoflavone that is commonly found in legumes, particularly in soy. Only a fraction of the human population (~50%) is able to convert daidzein into equol (which can then be further sulfated and glucuronated).^26,27^ The ability to produce equol presumably depends on the composition of a person’s microbiome and might be crucial for the health benefits that have been linked to soy isoflavones. At least two HuMet participants have this ability, but only for one of them was equol glucuronide detected in both blocks of the study, suggesting that (out of the two) only this person was exposed to soy (or other daidzein-containing food) before each of the study blocks.

Inspecting the list of metabolites with similar washout-like trajectories to 3-methylhistidine, we found five metabolites that have not been reported as dietary biomarkers. Interestingly, three out of these five metabolites are lipids that contain a C14 fatty acid residue (2-myristoyl-GPC [14:0], d_Fréchet_ = 0.5415; PC aa C32:2 [mainly consisting of PC [14:0_18:2]^12^], d_Fréchet_ = 0.5772; and lysoPC a 14:0, d_Fréchet_ = 0.5909). The steady decline of these metabolites over the two blocks, when participants were only exposed to the highly standardized challenge drinks, suggests that dietary choices (but not acute fasting status or macronutrient composition of the challenge drinks) modulate the levels of these complex lipids.

Taken together, this use case demonstrates the value of the similarity search as implemented in the selection module to depict metabolites showing the same dynamic behavior.

### Showcase platform comparison: Compare metabolites across metabolomics platforms

HuMet samples were profiled using a variety of metabolomics platforms, considering that no single approach can cover all parts of metabolism in sufficient quality.^28^ While their coverage of metabolites is mostly complementary, comparable measurements are available from the Metabolon HD4 platform and the Biocrates p150 kit for various amino acids, acylcarnitines, and glycerophospholipids. In this use case, we were interested in to what extent measures for matching metabolites correlate between the platforms. This comparison is of particular interest for matching metabolite pairs where the platforms do not quantify the exact same analytes due to the different measurement techniques. As an example, the 43 matching metabolite pairs listed in Yet et al.^29^ for Biocrates p150 (t-MS) and a prior version of Metabolon HD4 (nt-MS) include the pair H1 (hexose) (t-MS)/glucose (nt-MS). While the non-targeted technique measures the (relative) abundances of glucose, the most abundant hexose in human blood,^30^ the targeted assay measures the concentrations of all hexoses as a sum.

**Figure 4.**
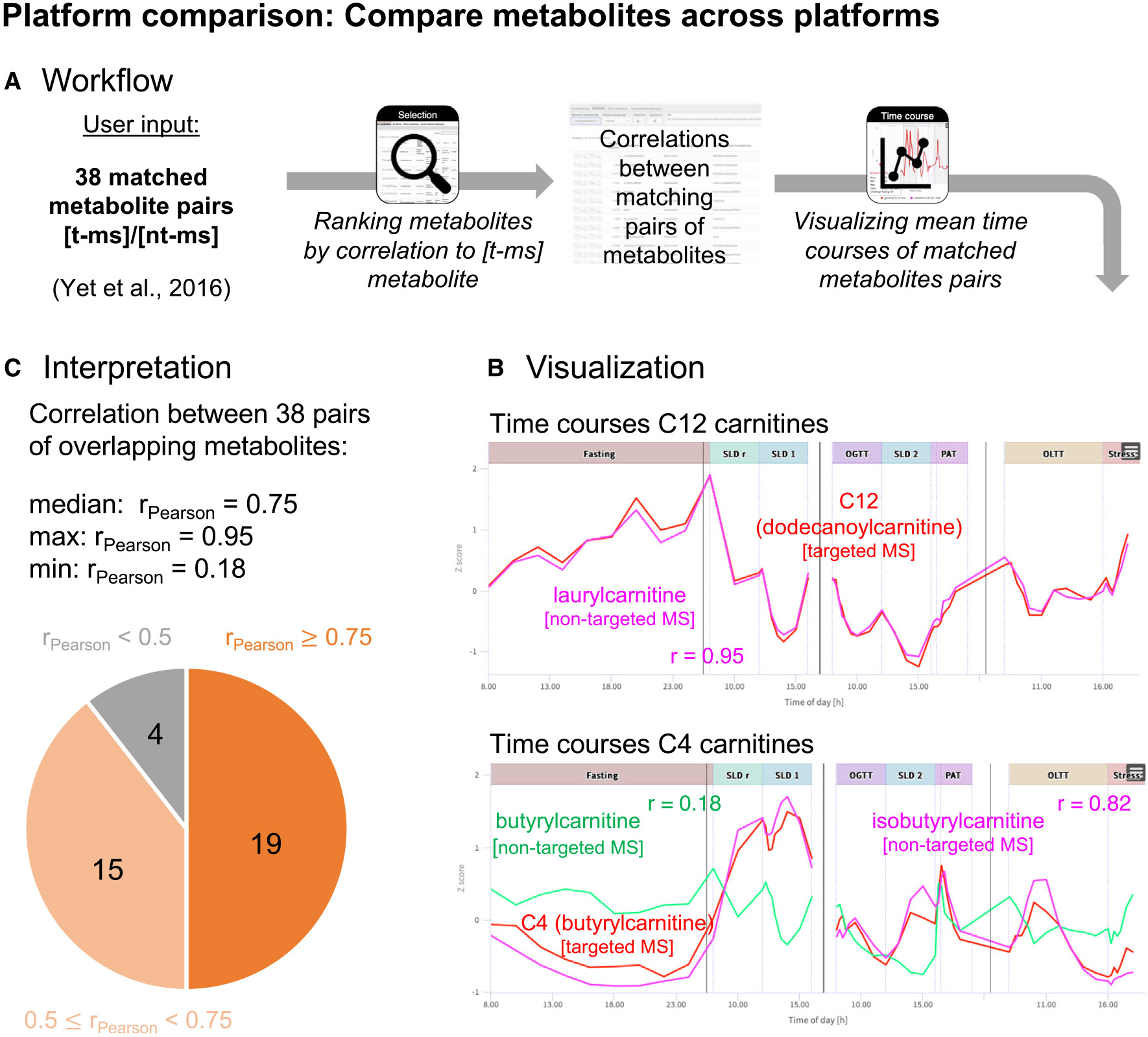
Comparison of measurements from different platforms (A) Workflow to explore the concordance of measurements for 38 pairs of matching metabolites from the non-targeted (Metabolon HD4) and targeted (Biocrates p150) platform (pairs taken from Yet et al.^29^). Pearson correlations of metabolites (across all time points and all subjects’ individual metabolite curves) are provided through the similarity search implemented in the module Selection. (B) Trajectories of the pair with strongest correlation (laurylcarnitine [P, nt-MS]/C12 [dodecanoylcarnitine] [P, t-MS]) with r = 0.95 and the pair with weakest correlation (butyrylcarnitine [P, nt-MS]/C4 [butyrylcarnitine] [P, t-MS]) with r = 0.18 are shown as displayed within the time course module. In the case of the C4 carnitines, the measurements for the isobaric isobutyrylcarnitine (P, nt-MS), which is added to the time course plot, showed a much stronger correlation with the C4 measurement from the targeted platform, indicating that C4 (butyrylcarnitine) (P, t-MS) and/or its dynamic changes might be dominated by the isoform isobutyrylcarnitine. (C) Overall, the concordance of measurements from the two platforms is high, with a median correlation of measurements of r = 0.75 and only four out of 38 pairs with correlations below 0.5.

Out of the 43 metabolite pairs, data on 38 pairs are available in the HuMet dataset. Overall, we observed a high correlation of measurements for the investigated metabolite pairs across the two platforms in HuMet, with a median correlation of 0.75 (Figure 4; Table S6). Also, glucose (nt-MS) and H1 (hexose) (t-MS) measurements were highly correlated (r = 0.87), which is in line with measured plasma hexose consisting mostly of glucose in humans. Only four pairs showed comparably weak correlations (r < 0.5) (Figure 4). In particular, the acylcarnitine measures butyrylcarnitine (nt-MS)/C4 (butyrylcarnitine) (t-MS) (r = 0.18) and glutarylcarnitine (nt-MS)/C6-OH (C5-DC) (t-MS) (r = 0.18) showed differences between the platforms. In the first case, the reason for this difference could be that the Biocrates p150 measure labeled as C4 (butyrylcarnitine) includes the isobaric isobutyrylcarnitine, while these two metabolites are measured as two separate analytes on the Metabolon HD4 platform. Correlation analysis of metabolite isobutyrylcarnitine (nt-MS)/C4 (butyrylcarnitine) (t-MS) (r = 0.82) indicated that C4 (butyrylcarnitine) (t-MS) and/or its dynamic changes might indeed be dominated by isobutyrylcarnitine. This is of particular interest as butyrylcarnitine and isobutyrylcarnitine derive from two fundamentally different pathways linked to the degradation of fatty acids and branched-chain amino acid, respectively. A similar scenario can be assumed to underlie the low correlations between glutarylcarnitine measured on Metabolon HD4 and the analyte labeled as C6-OH (C5-DC) measuring glutarylcarnitine and hydroxyhexanoylcarnitine together using the Biocrates p150 kit.

Taken together, this use case demonstrates the value of the HuMet Repository for comparing measurements from different metabolomics platforms and shows how these measurements can inform each other when time-resolved data are available for the same participants.

### Showcase systemic metabolic responses: Reveal and compare systemic responses to challenges

In this use case, we seek to answer the following questions: (1) “which areas of metabolism change after extended fasting compared to standardized overnight fasting in the reconstructed metabolic network?” and (2) “how do metabolic responses in particular pathways compare between three different nutritional challenges?” Using the inferred metabolic networks within the repository’s networks module, we can visualize and depict time-dependent responses to metabolic challenges in a metabolism-wide manner.

**Figure 5.**
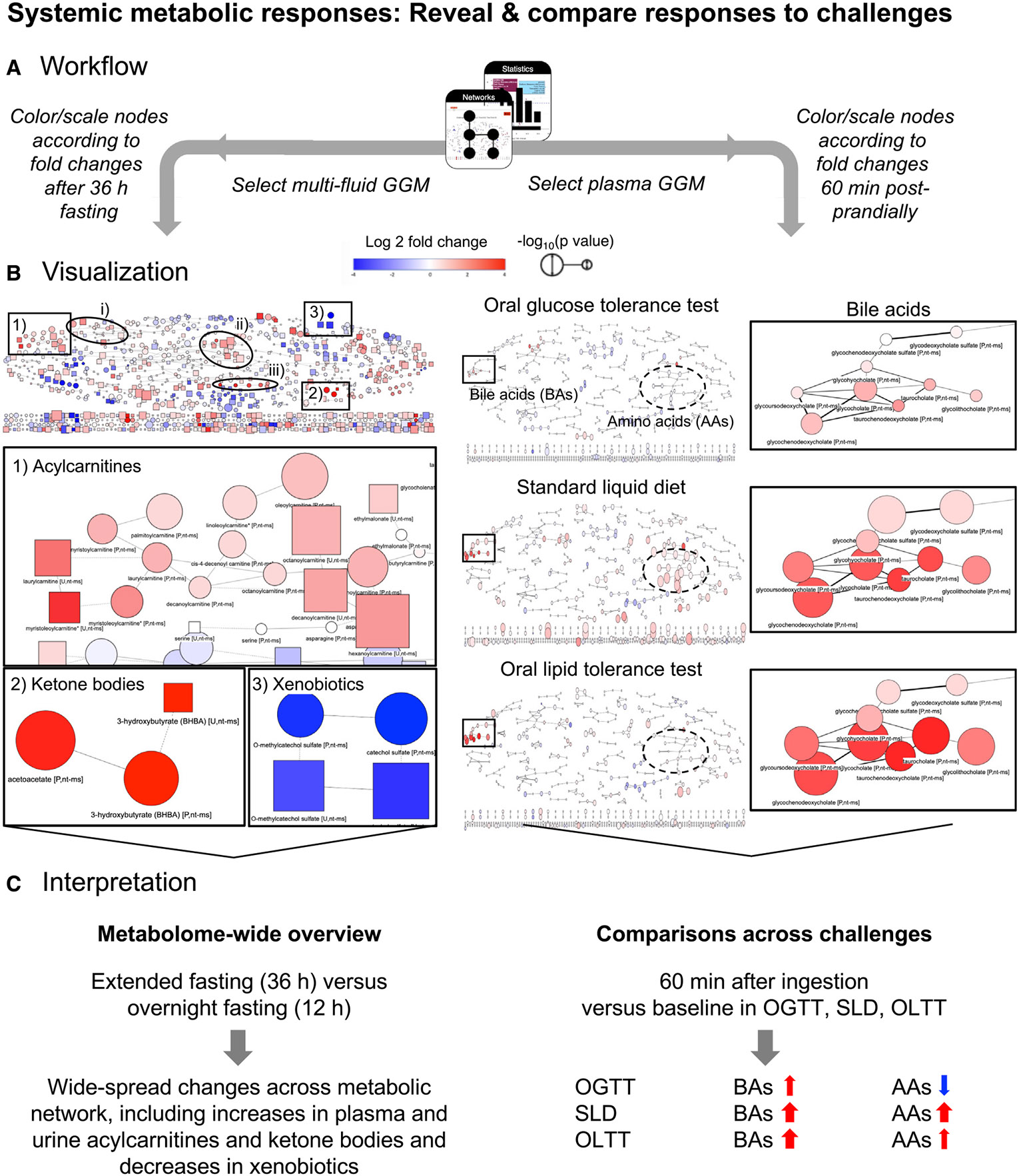
Contextualization of metabolic responses to challenges within reconstructed metabolic networks (A) Workflow to explore metabolite responses to challenges from a holistic, metabolome-wide perspective. To get an overview of changes after extended fasting (36 h) compared to overnight fasting (12 h), we select the multi-fluid metabolic network derived from the non-targeted metabolomics data in plasma and urine provided in the networks module (left). For a comparison of metabolite changes within particular pathways (here: bile acids and amino acids) 60 min after ingestion of the challenge drink, we select the single-fluid network generated based on plasma levels of the non-targeted (Metabolon HD4) and targeted (Biocrates p150) platform (right). To visualize responses, we map the log2 fold changes and *p* values resulting from t tests comparing metabolite levels after the respective challenge with the corresponding baseline levels (statistics module). Color displays the log2 fold change between challenge baseline and chosen time point, with red indicating an increase in metabolite concentration and blue indicating a decrease. Node size depicts the −log10 *p* value of changes between challenge baseline and the chosen time point. Thereby, node size increases with a lower *p* value. (B) Coloring and scaling of metabolite nodes according to changes after extended fasting of 36 h (versus overnight fasting [12 h]) shows that various parts of human metabolism are affected by adaptations to this challenge (left). This includes pathways such as beta-oxidation of fatty acids (indicated by increasing acylcarnitine levels in blood and urine; see zoom in for box 1) as well as the generation of ketone bodies (indicated by their increased levels in both fluids; see zoom in for box 2). Moreover, increases in metabolite levels are observed in further clusters delineated through circles (i: sulfated bile acids [and steroids]; ii: nucleotides [xanthine, hypoxanthine] and metabolites of the citrate cycle [malate, fumarate]; iii: dicarboxylic fatty acids [mainly C10–C18]; no zoom ins provided). Decreases (blue color) are seen for various pathways of xenobiotic metabolites, including benzoate metabolites (see zoom in for box 3). Coloring the network based on plasma metabolites by changes 60 min after ingestion of the challenge drinks (right) revealed a cluster of bile acids that similarly increased in SLD and OGTT and, to a lesser extent,. also in response to OGTT. In contrast, a cluster containing most amino acids showed decreases in OGTT and increases in SLD and OLTT, with less of an effect observed in the latter case (though 65% of the same protein mix was ingested with the OLTT drink as with the SLD drink). (C) Using the reconstructed metabolic networks helps to get a metabolome-wide overview of dynamic metabolic changes in response to specific challenges and facilitates comparison of effects between them.

To get a global view of changes in metabolism after prolonged fasting, we chose the multi-fluid network (Metabolon HD4 plasma and urine) with default cutoffs for the underlying partial correlation. On this backbone, we mapped statistical results comparing metabolite levels after extended fasting (36 h; TP 10 [see Table S7]) with levels after standardized overnight fasting (12 h; TP 1). In the resulting network, we saw wide-spread metabolic changes with prominent increases (indicated by a red color with high saturation and large circle sizes of metabolite nodes) in various pathways, including a metabolite cluster containing ketone bodies (and their precursors from ketogenic amino acid degradation) and a cluster containing acylcarnitines (Figure 5, left). Further increases were seen in clusters containing (1) sulfated bile acids (and steroids), in particular the monohydroxy bile acid derivative taurocholenate sulfate in blood and urine; (2) nucleotides (xanthine, hypoxanthine) and metabolites of the citrate cycle (malate, fumarate), which also increased during exercise; and (3) dicarboxylic fatty acids (mainly C10–C18) (indicated by circles in Figure 5B, left). While most of the metabolites in these clusters were also significantly higher after overnight fasting (e.g., acylcarnitines), increases in acetoacetate were only observed after prolonged fasting. Moreover, levels of sebacate (decanedioate) and taurocholenate sulfate were lower after overnight fasting compared to a fed state (2 h after SLD) but higher after prolonged fasting. The branched-chain amino acids leucine and isoleucine and several of their degradation products showed a similar behavior (Table S8). Most decreases (blue color) were observed in clusters containing xenobiotic metabolites or metabolites that have been linked to the human gut microbiome (Figure 5).

For comparison of metabolic responses across challenges, we selected a single-fluid GGM (Metabolon HD4 Plasma, Biocrates p150 plasma, and in-house biochemistry, partial correlation ≥ 0.12). Here, we mapped metabolic changes obtained for the OGTT (60 min versus baseline), SLD (60 min versus baseline), and OLTT (60 min versus baseline) and focused on two modules that exhibited consistent and different changes across challenges, respectively (Figure 5, right): (1) a cluster containing different bile acids consistently increased after 1 h in all three challenges and (2) a cluster containing various amino acids that showed considerably different responses between challenges; the majority of metabolites within this cluster decreased 60 min after glucose ingestion in the OGTT, while they increased after ingestion of the SLD drink and less extensively also after ingestion of the lipid-rich OLTT challenge drink. More detailed inspection of the bile acid time courses using the statistics module confirmed that for five (glyco- and taurocholate, glyco- and taurochenodeoxycholate, and taurodeoxycholate) of the bile acids in the cluster, the observed increases were statistically significant not only 1 h after ingestion of the lipid-containing SLD and OLTT challenge drinks but also 1 h after ingestion of glucose in the OGTT, with even higher fold changes observed after 15 and 30 min. Displaying the individual time courses of these bile acids across challenges in all participants using the time course module also showed that relative abundances and maximal fold changes of these bile acids strongly vary between individuals, challenge drinks, and even different contexts. When the SLD challenge drink was ingested “for lunch” 4 h after ingestion of the OGTT drink (SLD2), the observed maximal log2fcs for the five bile acids were almost as high as in the OLTT (log2fc = ~3–4); the same maximal log2fcs were seen when the SLD drink was ingested in the morning at 8 a.m. after prolonged fasting (SLDr). In contrast, when the SLD drink was provided “for lunch” 4 h after the first SLD drink in the morning on day 2 (SLD1), the maximal fold changes were smaller (log2fc = ~2; similar to the bile acid fold changes after OGTT), as the bile acid levels had not returned to the morning levels after these 4 h.

Taken together, this use case demonstrates the usefulness of the HuMet Repository to explore and compare metabolic responses in the context of metabolic networks and across challenges and pathways.

## DISCUSSION

The HuMet Repository described herein provides an easily accessible and explorable reference for metabolic responses to physiological challenges in healthy male individuals. The contained time-resolved metabolomics dataset is wide ranging regarding its metabolite coverage, with data from multiple different platforms, together capturing most areas of human metabolism in blood and urine. In particular, the non-targeted metabolomics data, which we added to the data that already existed from the HuMet study, increased the breadth of enclosed metabolic pathways (expanding from amino acids, glycerophospholipids, and lipoproteins to all major pathways) and their resolution (e.g., through the addition of complex lipid measures with resolved fatty acid chains). These extensions enable analyses of dynamic metabolic responses for metabolites in plasma and urine, for which the availability of such data has been previously limited. Thus, these additional data substantially enhance the comprehensiveness and granularity of time-resolved metabolomic readouts available for exploring human metabolism under challenge.

To bring the rich dataset to the scientific community, our repository goes beyond the idea of sharing data for reanalysis by experts according to the Findable, Accessible, Interoperable, and Reusable principles: by offering interactive exploration and visualization tools, we enable users to query the data directly and flexibly while taking the burden of data handling for these complex, high-dimensional data (15 subjects × 56 TPs/6 challenges × 2,656 metabolites/8 platforms × 4 biofluids) from them. As a consequence, answering *ad hoc* questions like “how robust is the metabolite that I identified as a disease biomarker in fasting versus non-fasting conditions?”, “how do dynamic changes in blood compare to those in urine for my metabolite of interest?”, “how individual is the metabolic response to exercise among healthy subjects for this metabolite?”, and “which metabolites exhibit the same longitudinal patterns over a challenge or the complete study duration?” is only a matter of clicks in the HuMet Repository, making the data thus more accessible for researchers with various backgrounds.

In addition to serving as a reference regarding specific metabolites of interest, the HuMet Repository facilitates systematic explorations into metabolic responses across biochemical pathways, physiological challenges, and metabolomics platforms as we demonstrated in the three showcases. First, we identified metabolites with washout-like trajectories by applying the built-in function for extracting metabolites with similar temporal profiles. Second, we demonstrated how assessing the similarity of metabolite trajectories can be used to compare readouts from different metabolomics platforms available for the same samples in the HuMet Repository. In contrast to previous work, where we used HuMet data for similar purposes,^12,31^ we here performed data analyses solely using the functionality of the HuMet Repository, without any additional effort for data processing, analysis, or visualization. Third, we highlighted the value of the repository’s data-derived metabolic networks to compare and contextualize statistical results from metabolome-wide analyses across six different challenges. Unlike knowledge-based metabolic networks such as KEGG,^18^ which typically omit many of the measured metabolites, the data-derived networks represent the entire set of metabolites measured in HuMet. In the present study, we demonstrated that partial correlations calculated from the HuMet study, encompassing time-resolved data from only 15 individuals, yielded networks in which functionally related metabolites were grouped similarly to those in networks derived from large cross-sectional data of more than 1,000 individuals.^14-16^ The networks reconstructed based on HuMet data are, thus, adequate to provide metabolic context for the global exploration of challenge-induced temporal changes.

Besides demonstrating the general applicability of the HuMet Repository, our showcasing analyses also derived concrete biological hypotheses. In our first case, we identified potential markers of dietary intake by extracting metabolites that indicated the exposure of our participants prior to the start of the two study blocks. Most of the metabolites that showed similar washout-like kinetic patterns as a known marker (3-methylhistidine) of a known prior exposure (meat)^23^ were dietary biomarkers of further food items, which were contained in the meal that was served to every participant at the evening prior to the study blocks or otherwise consumed before (e.g., metabolites of meat, garlic/onion, coffee, and soy). Interestingly, our analysis additionally revealed various phosphatidylcholines containing a C14:0 saturated fatty acid residue that showed the typical washout-like kinetic pattern for most participants. These metabolites are not listed as dietary biomarkers in FoodDB^24^ or Exposome-Explorer^25^ and are typically considered to be endogenous. Nonetheless, our results suggest that dietary choices strongly influence the blood levels of these metabolites. As bovine milk fat is rich in C14:0^32,33^ and blood C14 fatty acid abundance associates with habitual dairy intake,^34,35^ the steady decrease of the C14:0 phosphatidylcholines within each of the two study blocks might reflect the washout of metabolites originating from cream, which was an ingredient of the served dinner. On the other hand, considering previous results from Altmaier et al., who reported an association of phosphatidylcholines with shorter fatty acid chains (<C20) and higher saturation with fiber intake,^36^ the observed effect of constantly decreasing levels of C14:0 phosphatidylcholines could also be related to the lack of fiber in the provided challenge drinks.

Results from our second showcase emphasize that the concordance of measurements from different metabolomics platforms for the “same” (matching) metabolites can vary depending on sampling time and conditions. On average, we saw a high correlation of measurements across the investigated metabolites from the targeted Biocrates p150 and the non-targeted Metabolon platforms, despite differences in what exactly is quantified for matching compounds between the two platforms by design (e.g., relative abundance of glucose [non-targeted] versus absolute concentration of all [isobaric] hexoses [targeted]). We thereby replicated results from previous cross-platform studies, which indicated that these measures can be used largely interchangeably in cross-sectional data.^29,37^ At a first glance, the measures labeled with butyrylcarnitine were an exception. In the time-series data of the HuMet study, we only found a weak correlation of the targeted butyrylcarnitine analyte (representing the sum concentrations of isobaric C4 carnitines) with the non-targeted butyrylcarnitine measure. However, the correlation of targeted butyrylcarnitine with isobutyrylcarnitine measured as a separate analyte on the non-targeted platform was strong. Thus, when dynamically monitoring targeted C4 carnitine concentrations over challenges, the concentrations resemble changes in isobutyrylcarnitine, which is linked to the degradation of branched-chain amino acids, rather than butyrylcarnitine, which is derived from the beta-oxidation of fatty acids. In contrast, under overnight fasting conditions in cross-sectional data of 1,001 subjects, the correlation of targeted and non-targeted butyrylcarnitine measures was strong. Also, the association of measured levels with a genetic variant in the ACADS locus, which encodes an enzyme converting butyrylcarnitine, was identified independent of the platform.^29^ Hence, unlike its non-fasting concentrations, the fasting C4 concentrations in the targeted analysis indeed reflected the inter-individual differences in butyrylcarnitine levels.

In our third showcase, investigating the effects of prolonged fasting using the repository’s network visualization and statistics functionality, we found that most measured metabolites in plasma and urine were affected by the fasting challenge to some extent. As expected from the lack of other energy sources, the largest increases could be seen in metabolite clusters containing ketone bodies (and their precursors) and acylcarnitines, indicating the burning of fat.^38,39^ We also observed large increases in metabolite clusters containing dicarboxylic fatty acids (mainly C10–C18), indicating fat degradation through peroxisomal fat oxidation. This process involves microsomal omega-oxidation and is known to occur during fasting,^40^ in particular when mitochondrial beta-oxidation is impaired as, for example, in specific rare monogenic diseases.^41^ The produced dicarboxylic acids have been suggested as regulators of beta-oxidation,^42^ with a potential role in hepatic lipid accumulation induced by fasting.^40^ Hepatic lipid accumulation and steatosis have been linked to not only starvation conditions but also (chronic) excess of fatty acid influx into liver, as in many cases of obesity and type 2 diabetes.^43^ While the HuMet participants were healthy and non-obese, we observed increases in these dicarboxylic acids also when there was an (acute) excess of fatty acids after ingestion of the high-fat challenge drink for the OLTT (e.g., octadecanedioate log2fc = 1.45 [fasting] and 1.02 [OLTT]), matching the hypothesis of dicarboxylic acids being mediators of lipid accumulation. Interestingly, in our study, we observed increases in dicarboxylic acid levels also after the PAT (e.g., octadecanedioate log2fc = 0.90 [PAT]), which might provide an explanation for why prolonged physical exertion carries the risk of liver damage.^44^

Another class of metabolites that increased during extended fasting comprised sulfated bile acid as well as sulfated steroid derivatives (e.g., taurocholenate sulfate, glycochenodeoxycholate sulfate, dehydroepiandrosterone sulfate). All these compounds are products of a sulfation reaction catalyzed by the enzyme sulfotransferase 2A1; common genetic variants in the encoding gene SULT2A1 have been reported to influence the blood levels of these compounds.^21,45^ Despite various studies, the role of sulfotransferases in metabolic homeostasis is not fully understood yet and warrants further research.^46^

### Limitations of the study

While the showcases demonstrate the usefulness of the HuMet Repository, the data and our explorative approaches also have their limitations. First, the study was based on the idea of having a group of participants as similar as possible. This was realized with only 15 participants, all male, young, and of normal weight, with only minimal variations in BMI. The small sample size clearly restricts the statistical power to detect more subtle changes in metabolite levels, for example, as expected in response to the cold stress test. Also, the small size and restriction to young men lead to a lack of diversity in the study group, which, in turn, impedes the transferability of results to women or other age groups. However, the homogeneity of the group allows for analyzing inter-individual variation in metabolite levels and metabolic responses to the challenges in the absence of major sources that usually cause variation in metabolite levels, such as sex,^47,48^ age,^49-51^ and BMI.^52^ Second, while the availability of data on six different challenges for the same participants facilitates comparisons across challenges, all participants were exposed to these challenges in the same preset order. Therefore, we cannot exclude carry-over effects between challenges or study-specific overlay with diurnal variation of metabolites. Also, the standardized meals do not reflect the complexity and variety of everyday diets. As a consequence of the specific design of the study and its small sample size, the data in the HuMet Repository, while being representative of normal metabolic dynamics in response to the specific challenges, cannot be interpreted as representative of normal metabolic states. Nonetheless, the standardization and specific block-wise design of the HuMet study enabled the discovery of metabolites of prior exposure in the washout phase that are more difficult or impossible to pinpoint in studies that have only one TP or performed only one challenge under standardized conditions. Third, the comprehensive coverage of metabolites mainly through non-targeted approaches comes with the downside that only relative abundances of metabolites are reported, as opposed to absolute concentrations derived by the targeted methods. Consequently, our resource cannot provide “normal concentration ranges” for most measured metabolites, limiting the repository’s application as a quantitative reference. Nonetheless, information on the normal timing of challenge responses or the extent of a change in relation to the variation in the other challenges or TPs within individuals is less dependent on the type of quantification, as we confirmed through platform comparisons. Therein, temporal profiles of absolute and relative abundances of the same metabolite were highly concordant for metabolites where both measurements were available. Therefore, comparisons of timing/relative extent of responses between the repository and data from separately profiled samples (e.g., of patients in future studies) will be useful to identify responses that diverge from normal response patterns in these aspects. Fourth, as most data have been acquired from commercial metabolomics platforms, we do not have the rights to share the raw spectra for each measurement publicly. However, for various large epidemiological cohorts, cross-sectional datasets are available from the same metabolomics platforms, which enables the direct cross-linking of HuMet results to results from metabolome- and genome-wide association studies.^21,53-56^ Finally, explorative data analysis, as supported by our repository, can only be used for the generation of hypotheses, which need to be followed up by more specific and sophisticated data analyses and subsequent experiments.

### Conclusion

In conclusion, the HuMet Repository, freely accessible at https://humet.org, opens avenues for researchers with different backgrounds to explore human metabolism under challenge conditions. With its comprehensive coverage of the human metabolome in plasma and urine, its time-resolved metabolite profiles after six different metabolic challenges, and its interactive analysis and visualization tools, this repository facilitates explorative as well as systematic data analysis to identify dynamic metabolic responses in a metabolome- and “challenge-wide” fashion. Without the need to reprocess the data, researchers can leverage the repository to tackle question beyond those addressed in this work, e.g., how much does the coupling of kinetic behavior across metabolites differ when comparing responses between different challenges, and many more options, e.g., for cross-linking metabolite measures from different analytical platforms or estimating the stability of metabolite levels within individuals. Moreover, as we used highly standardized challenges for testing lipid, fasting, or exercise tolerance, they can be repeated in future studies, for instance, in studies involving women as well as different age groups and ethnicities. Adding the corresponding datasets to the repository as they become available will help to reflect dynamic metabolic changes in healthy individuals more broadly. Ultimately, data derived from these standardized challenge tests in patient cohorts could be directly compared to data in our repository for identifying deviations from the normal, healthy response. Therefore, the HuMet Repository could help unlock the full potential of standardized challenge tests and their metabolic readouts to identify metabolic aberrations when they are not yet visible in the rested, unperturbed state, thereby enabling new concepts for disease prevention or early diagnosis.

## STAR★METHODS

### RESOURCE AVAILABILITY

#### Lead contact

Further information and requests for data and code should be directed to and will be fulfilled by the lead contact, Gabi Kastenmüller (g.kastenmueller@helmholtz-muenchen.de).

#### Materials availability

This study did not generate new unique reagents.

#### Data and code availability

- Non-targeted metabolomics data have been deposited at MetaboLights and are publicly available as of the date of publication. Accession number is listed in the key resources table.
- All original code has been deposited at github.com/zenodo and is publicly available as of the date of publication. DOI is listed in the key resources table.
- Any additional information required to reanalyze the data reported in this paper is available from the lead contact upon request.

### EXPERIMENTAL MODEL AND STUDY PARTICIPANT DETAILS

#### HuMet study population

The present work is based on samples of the Human Metabolome (HuMet) study conducted at the Human Study Center of the Else-Kröner-Fresenius Center of Nutritional Medicine at the Technical University Munich. All details on study design, population, and existing data have been described previously.^7^ Briefly, 15 healthy male participants were recruited for the study. Participants were young (mean age of 27.8 years ±2.9), had normal weight (mean body mass index (BMI) of 23.1 kg/m^2^ ± 1.8), did not take any medication, and did not show any metabolic abnormalities.

#### HuMet study design

All participants underwent a series of six metabolic challenges within two 2-day test blocks (Figure 1). Twenty-four hours prior to each test block, participants were asked not to consume alcohol or engage in strenuous physical exercise. Participants were provided with the same meal (standard size, chicken-based with vegetables (FRoSTA Tiefkühlkost GmbH, Hamburg, Germany)) at 7 p.m. one day prior to each test block. During each study block, participants stayed within the study unit to reduce perturbation by environmental influences. Samples were collected at up to 56 time points in different intervals (every 15–240 min) over the study days depending on the collected biofluid (plasma, spot urine, exhaled breath condensate samples, breath air) and the particular challenge (Table S7).

Challenges covered extended fasting, ingestion of three different drinks with unique macronutrient compositions, a physical activity, and a stress test: (i) The fasting challenge consisted of a 36-h fasting period (from the dinner before block one until 8 a.m. on day two in the first block). During the challenge, participants drank 2.7 L of mineral water based on a defined drinking schedule. (ii) A standard liquid diet (SLD) drink was ingested at three occasions: SLDr – for “breakfast” on day two to recover from extended fasting, SLD1 – for “lunch” on day two, and SLD2 – for “lunch” on day three in the second block. The SLD drink consisted of a defined fiber-free formula drink (Fresubin Energy Drink Chocolate, Fresenius Kabi, Bad Homburg, Germany), providing one-third of the daily energy requirement of each participant. (iii) The oral glucose tolerance test (OGTT) on day three (block two) consisted of a 300 mL solution with mono- and oligosaccharides, equivalent to 75 g glucose after enzymatic cleavage (Dextro O.G.T., Roche Diagnostics, Mannheim, Germany). (iv) The oral lipid tolerance test (OLTT) on day four combined two parts of the SLD and one part of a fat emulsion containing predefined long-chain triglycerides (Calogen, Nutricia, Zoetemeer, Netherlands), while adjusting volumes per participant to provide 35 g fat/m^2^ body surface area. All challenge drinks were served at room temperature for ingestion within 5 min. (v) For the physical activity test (PAT) participants performed a 30 min bicycle ergometer training at a power level corresponding to their individual anaerobic threshold. (vi) In the cold stress test, participants were triggered by immersing one hand, up to wrist level for a maximum of 3 min in ice water. For a complete protocol of the challenge procedure and the collection of samples, see Krug et al..^7^

The ethical committee of the Technische Universität München approved the HuMet study protocol (#2087/08), which is in correspondence with the Declaration of Helsinki.

### METHOD DETAILS

#### Non-targeted metabolomic profiling

In this study, we acquired non-targeted metabolomics data by profiling plasma and urine samples of the HuMet study on the Metabolon HD4 platform using liquid chromatography coupled to mass spectrometry (LC-MS) at Metabolon, Inc. (Durham, NC, USA). This platform applies four different analytical methods optimized for measuring metabolites with different physicochemical properties: (i) a reverse phase (RP)/ultra-high-performance liquid chromatography (UPLC)-MS/MS method with electrospray ionization (ESI) in positive mode optimized for hydrophilic compounds, (ii) an RP/UPLC-MS/MS with ESI in positive mode optimized for more hydrophobic compounds (iii) an RP/UPLC-MS/MS with ESI in negative mode, and (iv) a hydrophilic interaction liquid chromatography (HILIC)/UPLC-MS/MS with ESI in negative mode. All methods utilized a Waters ACQUITY UPLC and a Thermo Scientific Q-Exactive high resolution/accurate mass spectrometer (operating at a mass resolution (m/Dm) 35,000) interfaced with a heated electrospray ionization (HESI-II) source. For methods i – iii, a C18 column from Waters (UPLC BEH C18-2.1 × 100 mm, 1.7 μm) was used, method iv utilized a HILIC column (Waters UPLC BEH Amide 2.1 × 150 mm, 1.7 μm).

Sample processing for and analytical procedures of the Metabolon HD4 platform have been described in detail previously.^10^ Briefly, EDTA-plasma and spot urine samples, which were kept at −80°C until analysis, were first thawed. Then, several recovery standards, which were carefully chosen not to interfere with the measurement of endogenous compounds, were spiked into 100 μL of every sample to allow chromatographic alignment and to monitor instrument performance. For protein precipitation and metabolite extraction, samples were mixed with methanol under vigorous shaking for 2 min (Glen Mills GenoGrinder 2000). After centrifugation, the resulting extracts were split into five portions for each sample: four aliquots for analysis by the different LC-MS methods and one aliquot for backup. The extracts were placed briefly on a TurboVap (Zymark) to remove the organic solvent and then stored overnight under nitrogen. Before LC-MS analysis, the extracts were reconstituted in solvents compatible for the MS methods (with each reconstitution solvent containing a series of standards at fixed concentrations to ensure injection and chromatographic consistency). All described sample processing steps were automated using a MicroLab STAR system from Hamilton Company (Reno, NV, USA).

For LC-MS analysis by method i (acidic positive ion conditions), the extracts were gradient eluted from a C18 column (see above) using water and methanol, containing 0.05% perfluoropentanoic acid (PFPA) and 0.1% formic acid (FA). For analysis by method ii (acidic positive ion conditions), the extracts were gradient eluted from the same C18 column using methanol, acetonitrile, and water, containing 0.05% PFPA and 0.01% FA. For analysis by method iii (basic negative ion conditions), the extracts were gradient eluted from a separate C18 column using methanol and water with 6.5 mM ammonium bicarbonate at pH 8. For analysis by method iv (basic negative ion conditions), the extracts were gradient eluted from a HILIC column using a gradient consisting of water and acetonitrile with 10 mM ammonium formate at pH 10.8. The MS analysis alternated between full scans (covering 70–1000 m/z) and data-dependent MS^n^ scans using dynamic exclusion.

Peak identification and alignment from the recorded spectra, were performed using Metabolon’s in-house hardware and software. Metabolites were identified by comparison of the experimental spectra to entries in Metabolon’s in-house library, which was collected from the measurement of commercially available purified standards (~3,300 at time of analysis) or recurrent spectra from either named compounds (or classes), for which no authenticated standard was available (marked by a tag next to the metabolite name in Table S1), or from structurally unnamed biochemicals. Note that, for the present study, only the results from measuring named metabolites were purchased from Metabolon. The area-under-the-curve (AUC) of the peaks indicated as the *quantification ions* in the library entries were used to quantify metabolites. To account for differences in solute concentrations, raw peak AUC values of metabolite in urine were normalized by osmolality. Raw peak AUC values (plasma) and osmolality-normalized peak AUC values (urine) of each metabolite were additionally normalized to account for instrument inter-day tuning differences by dividing the values of each metabolite at each run day by the median of values for the metabolite on this day (i.e., setting the run day medians to one). Before data release, a series of manual curation procedures were carried out at Metabolon to remove metabolite signals representing system artifacts, mis-assignments, and background noise and to confirm the consistency of peak identification and quantification among the various samples. In cases where compounds were detected by more than one of the four analytical methods, the abundance of the compound was reported using the AUC from the method that showed the highest measurement quality according to the aforementioned quality control. This work was based on proprietary visualization and interpretation software.

As the focus of the HuMet study was on the dynamic changes of metabolite levels within individuals, samples from the same individual were measured on the same run day (plate) to the extent possible, leading to a run day design where the plasma samples of two participants were analyzed on three different run days while assigning samples of block 1 (days 1 and 2), samples of day 3 (block 2), and samples of day 4 (block 2) to the same run day, respectively. Within run days the order of samples was randomized. Due to the lower number of urine samples, the samples from all time points of two participants were measured on the same run day. Plasma and urine samples of subject 4 were measured in duplicates; for this subject, we used the mean of both measurements. Several quality control (QC) samples, which underwent the same sample processing as the HuMet samples, were measured spaced evenly among the experimental samples: Ultra-pure water samples served as process blanks; pooled matrix samples (CMTRX) generated from all HuMet samples (only for urine samples) and aliquots of a pool of well-characterized human plasma (MTRX4) (both for plasma and urine runs) served as technical replicates to assess process variability across run days of the analysis. Relative standard deviation of CMTRX (urine) and MTRX4 (plasma) measurements are provided in Table S1.

As a result of the analyses of 833 plasma and 240 urine samples on the Metabolon HD4 platform, relative abundances (normalized peak AUCs) are available for a total of 595 plasma and 619 urine metabolites. These metabolites were assigned to eight chemical classes termed super-pathways (amino acids, carbohydrates, cofactors and vitamins, energy, lipids, nucleotides, peptides, xenobiotics), each being divided into two or more subpathways, resulting in a total of 78 and 68 subpathways for the plasma and urine metabolites, respectively (Table S1).

#### Lipidomic profiling

Lipid concentrations in HuMet plasma samples of four participants were analyzed on the Lipidyzer platform of AB Sciex Pte. Ltd. (Framingham, MA, USA) by Metabolon Inc., Durham, NC, USA. Samples were kept at −80°C until analysis. The protocol of lipid quantification using this platform has been described in detail elsewhere.^12^ In brief, after thawing, lipids were extracted from the plasma samples with dichloromethane and methanol following a modified Bligh-Dyer extraction. For analysis, the lower, organic phase, which included internal standards, was used and concentrated under nitrogen. Extracts were reconstituted with 0.25 mL of dichloromethane: methanol (50:50) containing 10 mM ammonium acetate and placed in vials for infusion-MS analysis on a Sciex 5500 QTRAP equipped with a SelexION differential ion mobility spectrometry (DMS) cell, which allows separation of different (lyso)phospholipids [(lyso)phosphatidylcholines ((L)PCs), -ethanolamines ((L)PEs), -inositols (PIs)] and sphingomyelins (SMs). Extracts were analyzed using multiple reaction monitoring (MRM) in two sequential flow injection analysis (FIA) runs, alternating between positive and negative polarity. Free fatty acids (FFAs), tri- and diacyglycerols (TAGs, DAGs), ceramides (CERs), lactosyl-, hexosyl-, and dihydroceramides (LCERs, HCERs, DCERs), and cholesterylesters (CEs) were measured using separation through the DMS cell. Lipids were quantified relative to appropriate stable isotope labeled internal standards. Concentrations are provided in mmol/l. The Lipidyzer platform allowed for absolute quantification of 965 lipids distributed over 14 lipid classes: (CE, TAG, DAG, FFA, PC, PE, PI, LPC, LPE, SM, CER, HCER, LCER, DCER).

#### Integrating previous HuMet metabolomics data

The HuMet study samples were previously profiled on three different “in-house” and three different vendor-based platforms. For integration into our HuMet Repository, these data were used as published and provided in Krug et al..^7^

Measured metabolites from these six platforms and the analytical methods used were described in detail in Krug et al.^7^ and are only briefly summarized here: (i) “*In-house biochemistry*”: Standard biochemistry assays were used to assess blood levels of glucose, lactate, insulin, and non-esterified fatty acids (NEFA) in 840 plasma samples (15 subjects x 56 time points). Venous plasma glucose and lactate concentrations were profiled using an enzymatic amperometric technique, insulin was measured by ELISA, NEFA were quantified in plasma by an enzymatic colorimetric method. All assays were performed at the Technische Universität München. (ii) “*In-house FTICR-MS*”: Flow injection electrospray ionization ion cyclotron resonance Fourier transform mass spectrometry (FTICR-MS) measurements were performed at Helmholtz Zentrum München. A total of 201 mass spectral features from volatile compounds were reported in 55 breath condensates samples (5 subjects x 11 time points of the first block). (iii) *“In-house PTR-MS”*: Proton transfer reaction mass spectrometry (PTR-MS) was used to profile 341 breath air samples (11 subjects x 31 time points). Analyses were performed by researchers from Helmholtz Zentrum München and yielded 106 mass spectral features of volatile compounds. (iv) *“Biocrates p150”*: Absolute*IDQ* p150 kits from Biocrates Life sciences AG, Innsbruck, were used to perform flow injection analysis mass spectrometry (FIA-MS) of 840 plasma samples (15 subjects x 56 time points), yielding quantities for 132 blood metabolites after quality control. (v) *“numares”/“Chenomx”*: NMR spectra of 810 plasma samples (15 subjects x 54 time points) and 195 urine samples (15 subjects x 13 time points) were determined by numares (formerly LipoFit Analytic GmbH, Regensburg, Germany). For plasma samples, a total of 28 metabolites were identified by the company based on these spectra. For urine samples, only the levels of six metabolites were extracted from the spectra (at Helmholtz Zentrum München) using the software Chenomx NMR suite 7.0.

All data were used as preprocessed and provided in Krug et al.,^7^ unless stated otherwise in the following.

### QUANTIFICATION AND STATISTICAL ANALYSIS

#### Data preprocessing and transformations

Platform-specific quality control and normalization steps depend on the analytical method and are described in the respective paragraphs on data acquisition. Note that the platform-specific normalization steps taken to account for instrument drifts or inter-day tuning differences might not be sufficient to avoid all effects of batch-wise measurements on the results of downstream data analyses, in particular when data from multiple platforms are combined.

All quality controlled and normalized metabolomics data were forwarded to integration into the HuMet Repository. Thereby, metabolite names and abbreviations were kept as provided by the specific platforms. Metabolite identifiers within the repository contain the platform specific name, information on the fluid, in which they were measured (P: plasma; U: urine; BA: breath air; BC: breath condensate), and information on the platform (nt-ms: Metabolon HD4; t-ms: Biocrates p150; Lipidyzer: Lipidyzer; NMR: numares/Chenomx; PTRMS: In-house PTR-MS; ICR: In-house FTICR-MS; chem.: In-house biochemistry). Named metabolites were assigned to the eight different metabolite classes (“super-pathways”) as used for the Metabolon HD4 platform and to “subpathways” according to the categories given by the platforms. We manually annotated metabolites with links to compounds in knowledge-based platforms, including ChEBI,^19^ KEGG,^18^ PubChem,^63^ and HMDB.^20^ Through the ChEBI crosslinks, synonyms for metabolite names were incorporated.

For samples, information on the fluid, the subject (1–15), and the time point (1–56) (Table S7) are used for identification (some breath air measurement were between two time points as defined for plasma/urine; they are denoted by 10.5, 11.5, 27.5, 39.5; for the six NMR urine metabolites (ChenomX), a sample from an additional time point (57; day 4: 7 p.m.) was measured).

The following preprocessing steps and transformations were applied to all metabolomics data.

##### Manual curation

To identify and remove outliers/implausible values, we systematically filtered single data points whose log2-transformed values were outside the mean ± 4 times the standard deviation window for the particular metabolite and time point, while omitting data points from measurements within the first 30 min of a study challenge (to avoid deletion of biologically meaningful challenged-induced concentration peaks of subjects). As a result, we identified 163 outlier data points, of which 92 data points were excluded after manual inspection. This cleaned dataset is integrated into our repository and can be downloaded from the website.

##### Data transformations

In addition to the original concentration or relative abundance values, we provide the data after further transformations for display in the *Time Course* module: (i) *z-scores* based on the log2-transformed concentrations/relative abundances to facilitate comparisons across metabolites and platforms, (ii) *log2 fold changes (block)* calculated between the time points within each block relative to the first time point of the respective block, and (iii) *log2 fold changes (challenge)* calculated between the time points in a specific challenge and the challenge baseline (see Table S7).

##### Imputation

Some of the downstream statistical analyses used in the HuMet Repository, such as network inference with GGM, require a full dataset without missing values. Before imputation, the manually curated dataset was filtered for metabolites with less than 30% missingness (*n* = 493) across all samples measured on the particular platform (Table S1). Based on the filtered dataset, we imputed missing values using four different imputation methods: (i) Machine learning algorithm *missForest* (ntree = 1500, mtry = 22), which is implemented in the R package *missForest* (version 1.4). The algorithm is based on a random forest approach and imputes missing values by iteratively (maximum iterations = 10) predicting missing values using the available data.^58,64^ (ii) Predictive mean matching (PMM) using the parallelized mice function *futuremice* (method = “2lonly.pmm”, m = 5) implemented within the R package *mice* (version 3.15.0). The method facilitates temporarily consistent longitudinal imputation with PMM.^59^ (iii) Subject- and study block-specific linear imputation in cases where data points preceding and succeeding a missing value were available. Here, we used the *approx* function from the basic R package *stats* (version 4.2.3) facilitating the estimation of missing values based on linear trends. (iv) K nearest neighbor (KNN) based imputation using mt_pre_impute_knn (k = 10, method = “knn.obs.euc.sel”) from the R package *maplet*^60^ (version. 1.1.2). The method uses the KNN algorithm, pre-selecting a subset of metabolites correlated with the target metabolite with missingness.^65^

#### Statistical analysis/functionality

##### Metabolite time course similarity

We provide several distance measures (Fréchet, Euclidean, Manhattan) and Pearson correlation to rank metabolites according to their similarity in temporal profiles. All measures are calculated based on z-scored data and depend on user-selected settings such as the choices of subjects and time-range. The distance/correlation between the temporal curves of two metabolites is calculated within each subject first; subsequently, we calculate the average distance/correlation across all chosen subjects. We additionally provide Fréchet distance and Pearson correlation calculated based on the mean metabolite trajectories (mean *Z* score over all participants at each time point). The Fréchet distance (on average trajectories) is set to default within the similarity tool. It uses a window approach to search for the smallest distance between curves in a defined time frame. This time frame is defined as follows: Maximum of +/− 30 min within all challenges except the extended fasting. Within the extended fasting challenge, we allow for comparison of time-points within a range of +/− 120 min.

We used the *dist* function implemented within the R package *proxy* (version 0.4–23) to calculate the Euclidean and Manhattan distances. The R package *stats* (version 4.2.3) was used to calculate the Pearson correlation. To calculate the Fréchet distance we used the *distFrechet* function implemented within the R package *kmlShape* (version 0.9.5), a parallelized version of the *distFrechet* function of the R package *longitudinalData.*

##### Paired t-tests

We use paired t-tests to test for significant changes in metabolite levels between two time points based on the log2-transformed imputed or non-imputed (selectable by the user) concentrations/relative abundances, using the function *t.test* implemented in the R package *stats* (version 4.2.3). To adjust for multiple testing, we offer corrections based on the false discovery rate (FDR) by Benjamini-Hochberg^66^ (q < 0.05) or Bonferroni (*p* < 0.05/(n _metabolites_ *n_time points_)). The levels of adjustment are reactive to the number of metabolites and time points submitted to statistical analysis. The user can select the time range and the option whether only the last time point or all time points within the range are compared to the first time point. Results are visualized within a volcano plot by using the function *plot_ly* of the R package *plotly* (version 4.9.1). Each data point within the volcano plot can be colored by superpathway or metabolomics platforms.

##### Power calculation

We performed a power calculation to estimate the number of participants that would have been needed to detect metabolic changes in response to the cold stress test at the Bonferroni-corrected significance threshold (α = 0.05/2656 = 1.88e-5) with a power of 80%. We based the calculation on the effect expected for cortisol as cortisol levels (in saliva) have been shown to respond to a cold stress test in previous studies.^13^ As effect size in plasma, we used the Hedges corrected version of Cohen’s d (d_corrected_ = d * (n_subjects_ - 2)/(n_subjects_ - 1.25))^67^ calculated based on the change in cortisol plasma levels (log2-transformed) after 15 min in our study (*p-value* = 0.012). For power calculation, the *pwr.t.test* function of the *pwr* R package (version 1.3.0) was used. Under these assumptions, 58 (57.23) participants would have been needed to detect the change with 80% power at the selected α.

##### Network generation

Knowledge-based networks were constructed based on the annotated super- and sub-pathway structure of metabolites. This structure provides a quick overview of available metabolites from different platforms.

Network inference of Gaussian Graphical Models (GGMs) is based on partial correlations of metabolite concentrations/abundances (single fluid, imputed and log2-transformed data). These models have previously demonstrated to reconstruct biological pathways from cross-sectional metabolomics data derived from Biocrates and Metabolon platforms.^14^ To calculate partial correlations for the HuMet datasets we used the shrinkage estimator approach “GeneNet”, which is available within the R package *GeneNet* (version 1.2.14), choosing the “dynamic” method for estimation. This method relies on the function *dyn.pcor* implemented within the R package *longitudinal* (version 1.1.12), which takes the longitudinal data structure with repeated measurements from the same participant into account.^17^ If both dynamic partial correlation and Pearson correlation between two metabolites were statistically significant at a 5% significance threshold, pairwise metabolite connections were integrated into the network. Thereby, the user can choose between Bonferroni or FDR correction for multiple testing or restrict edges in the displayed network to those greater than several pre-defined dynamic partial correlation values.

Using this approach, we inferred and provide multiple single fluid networks based on one or more plasma or urine datasets from different platforms. For the generation of the multi-fluid network based on the plasma and urine datasets from the Metabolon HD4 platform, we merged the corresponding single fluid networks by connecting the same metabolites measured in plasma and urine by an additional edge, closely following the procedures reported in Do et al. for creating an overlaid network.^15^

### ADDITIONAL RESOURCES

#### Implementation of the web-based repository

The HuMet Repository is written in R^57^ using shiny, an R package that enables setting up web-based graphical user interfaces (GUIs) while allowing to execute R code on the backend,^62^ and supporting packages. All R packages used for building the interactive HuMet Repository are listed in the key resources table.

The repository loads the preprocessed data, metabolite information, sample information upon session start. Thereafter, the repository is reactive to the user’s choices of options. These include exclusion of data points due to selected time points, subjects, and platforms. The repository visualizes the chosen data in interactive plots that, e.g., provide additional information via hover-over functionality, allow for zooming, and data-dependent coloring of data points.

The datasets for this study can be found in the Download section of the HuMet Repository: https://humet.org.

Data from the non-targeted metabolomics platform is available at the MetaboLights Database: http://www.ebi.ac.uk/metabolights/MTBLS89.

## Supplementary Material

1

2

3

4

5

6

7

8

## Figures and Tables

**Table 1.** Overview of metabolomics data provided within the HuMet Repository

Medium	Platform	Subjects	Time points	Metabolites	Main pathways	Unit	Reference
Plasma	Metabolon HD4 (nt-MS)	15	56	595	amino acids, peptides, carbohydrates, energy, cofactors and vitamins, lipids, nucleotides, xenobiotics	norm. ion counts^a^	this study^b^
Plasma	Lipidyzer (Lipidyzer)	4	56	965	lipids	μmol/L	this study^c^
Plasma	Biocrates p150 (t-MS)	15	56	132	amino acids, lipids	μmol/L	Krug et al.^7^
Plasma	Numares (LipoFIT) (NMR)	15	54	28	lipoproteins	μmol/L; nm; mg/dL	Krug et al.^7^
Plasma	in-house biochemistry (chem.)	15	56	4	–	mg/dL;	Krug et al.^7^
Urine	Metabolon HD4 (nt-MS)	15	16	619	amino acids, peptides, carbohydrates, energy, cofactors and vitamins, lipids, nucleotides, xenobiotics	norm. ion counts^d^	this study
Urine	Chenomx (NMR)	15	13	6	ketone bodies	μmol/mmol creatinine	Krug et al.^7^
Breath air	in-house PTR-MS	11	32	106	features of volatile compounds	norm. ion counts^e^	Krug et al.^7^
Breath condensate	in-house FTICR-MS	5	11	201	features of volatile compounds	norm. ion counts^f^	Krug et al.^7^

norm., normal; PTR, proton transfer reaction; FTICR, ion cyclotron resonance Fourier transform.

a Ion counts normalized by batch median.

b Data on selected time points have been part of a previous publication.^11^

c Data on selected metabolites from this platform (69 specific phosphatidylcholines) have been part of a previous publication.^12^

d Ion counts normalized by osmolality and batch median.

e Ion counts normalized to count rate of primaries and water clusters and change in transmission over time and converted to parts per billion volume (ppbv).

f Ion counts normalized on the sum of signal intensity per spectrum and subject-wise on the sum of normalized signal intensities.

**Table 2.** Metabolites with largest changes after each challenge

Challenge (period tested)	Metabolite with min/max fold change (platform)	Fluid (time point)	Log2 fold change	*p* value	Metabolite with lowest *p* value (platform)	Fluid (time point)	Log2 fold change	*p* value
Fasting (12–36 h)	3-methoxy-catechol sulfate (nt-MS)	urine (at 36 h)	−6.1	4.6e–8	catechol sulfate (nt-MS)	plasma (at 36 h)	−4.5	2.2e–14
Fasting (12–36 h)	3-hydroxy-butyrate (BHBA) (nt-MS)	urine (at 36 h)	7.7	2.8e–8	catechol sulfate (nt-MS)	plasma (at 36 h)	−4.5	2.2e–14
OGTT (0–2 h)	5-dodecenoate (12:1n7) (nt-MS)	plasma (at 90 min)	−1.6	9.2e–8	insulin (chem.)	plasma (at 30 min)	3.1	1.1e–11
OGTT (0–2 h)	insulin (chem.)	plasma (at 30 min)	3.1	1.1e–11	insulin (chem.)	plasma (at 30 min)	3.1	1.1e–11
SLD2 (0–3 h)	decanoylcarnitine (nt-MS)	plasma (at 2 h)	−1.8	6.9e–9	3-hydroxy-3-methylglutarate (nt-MS)	plasma (at 2 h)	2.0	1.7e–13
SLD2 (0–3 h)	taurochenodeoxy-cholate (nt-MS)	plasma (at 60 min)	3.5	2.1e–7	3-hydroxy-3-methylglutarate (nt-MS)	plasma (at 2 h)	2.0	1.7e–13
PAT (0–30 min)	palmitoylcholine (nt-MS)	plasma (at 30 min)	−3.4	1.3e–7	lactate (nt-MS)	plasma (at 30 min)	2.2	1.3e–9
PAT (0–30 min)	lactate (chem.)	plasma (at 30 min)	2.5	1.1e–8	lactate (nt-MS)	plasma (at 30 min)	2.2	1.3e–9
OLTT (0–4 h)	phosphate (nt-MS)	urine (at 4 h)	−5.5	2.9e–10	2-hydroxy-decanoate (nt-MS)	plasma (at 4 h)	1.6	1.7e–12
OLTT (0–4 h)	taurocholate (nt-MS)	plasma (90 min)	4.2	1.4e–7	2-hydroxy-decanoate (nt-MS)	plasma (at 4 h)	1.6	1.7e–12
Stress (0–30 min)	no significant changes							

**Table:** KEY RESOURCES TABLE

REAGENT or RESOURCE	SOURCE	IDENTIFIER
Deposited data		
Non-targeted metabolomics data	This paper	MetaboLights: MTBLS89
Software and algorithms		
R environment (version 4.2.3)	R project^57^	RRID:SCR_001905; RRID:SCR_003005
R package missForest (version 1.4)	Stekhoven et al.^58^	https://cran.r-project.org/web/packages/missForest/; RRID:SCR_018543
R package mice (version 3.15.0)	van Buuren et al.^59^	https://cran.r-project.org/web/packages/mice/
R package maplet (version 1.1.2)	Chetnik et al.^60^	https://github.com/krumsieklab/maplet/
R package proxy (version 0.4–23)	N/A	https://cran.r-project.org/web/packages/proxy/
R package kmlShape (version 0.9.5)	Genolini et al.^61^	https://cran.r-project.org/src/contrib/Archive/kmlShape/
R package longitudinalData (version 2.4.1)	N/A	https://cran.r-project.org/web/packages/longitudinalData/index.html
R package GeneNet (version 1.2.14)	Opgen-Rhein & Strimmer^17^	https://cran.r-project.org/web/packages/GeneNet/
R package longitudinal (version 1.1.12)	Opgen-Rhein & Strimmer^17^	https://strimmerlab.github.io/software/longitudinal/
R package pwr (version 1.3.0)	N/A	https://cran.r-project.org/web/packages/pwr
shiny (version 1.7.4)	Chang et al.^62^	https://cran.r-project.org/web/packages/shiny/; RRID: SCR_0011626
R package shinydashboard (version 0.7.2)	N/A	https://cran.r-project.org/web/packages/shinydashboard/
R package shinydashboardPlus (version 2.0.3)	N/A	https://github.com/RinteRface/shinydashboardPlus/
R package shinyWidgets (version 0.5.0)	N/A	https://github.com/dreamRs/shinyWidgets/
R package shinyBS (version 0.61)	N/A	https://cran.r-project.org/web/packages/shinyBS/
R package shinycssloaders (version 0.3)	N/A	https://cran.r-project.org/web/packages/shinycssloader/
R package DT (version 0.27)	N/A	https://cran.r-project.org/web/packages/DT
R package tableHTML (version 2.0.0)	N/A	https://cran.r-project.org/web/packages/tableHTML/
R package Highcharter (version 0.7.0)	N/A	https://jkunst.com/highcharter/
R package plotly (version 4.9.1)	N/A	https://cran.r-project.org/web/packages/plotly/index.html
R package visNetwork (version 2.0.9)	N/A	https://cran.r-project.org/web/packages/visNetwork/
R package igraph (version 1.4.1)	N/A	https://cran.r-project.org/web/packages/igraph/; https://doi.org/10.5281/zenodo.7682609
HuMet Repository code	This paper	https://github.com/SystemsMetabolomics/HuMet-Rep; https://doi.org/10.5281/zenodo.12758689
R package doParallel (version 1.9.15)	N/A	https://github.com/RevolutionAnalytics/doparallel/
R package openxlsx (version 4.2.5.2)	N/A	https://cran.r-project.org/web/packages/openxlsx/; RRID: SCR_019185
Other		
HuMet study (design, data)	Krug et al.^7^	N/A
HuMet Repository (online resource, data download)	This paper	https://humet.org/

## Highlights

- Time-resolved metabolomic responses to six physiological challenges in healthy individuals
- Subject-level response profiles for >2,600 metabolites measured in blood, urine, or breath
- Three showcases of data usage for exploring human metabolism under challenge conditions
- Interactive querying and visualization of response profiles and statistics at humet.org
