# Supplementary material for: The HuMet Repository: Watching human metabolism at work
Source: Cell Rep. Author manuscript; Available in PMC 2024 Oct 28. (PMC11513335; doi:10.1016/j.celrep.2024.114416)
Supplement: 7 [file NIHMS2019468-supplement-7.pdf]

Supplementary Table 7: Coding of time points

time pont	time of day	block	day	challenge
1	08:00	1	1	Fasting 12h
2	10:00	1	1	Fasting 14h
3	12:00	1	1	Fasting 16h
4	14:00	1	1	Fasting 18h
5	16:00	1	1	Fasting 20h
6	18:00	1	1	Fasting 22h
7	20:00	1	1	Fasting 24h
8	22:00	1	1	Fasting 26h
9	24:00	1	1	Fasting 28h
10	08:00	1	2	Fasting 36h/Fasting recovery 0h
11	10:00	1	2	Fasting recovery 2h
12	12:00	1	2	Standard liquid diet 0h
13	12:15	1	2	Standard liquid diet 15min
14	12:30	1	2	Standard liquid diet 30min
15	12:45	1	2	Standard liquid diet 45min
16	13:00	1	2	Standard liquid diet 60min
17	13:30	1	2	Standard liquid diet 90min
18	14:00	1	2	Standard liquid diet 120min
19	15:00	1	2	Standard liquid diet 3h
20	16:00	1	2	Standard liquid diet 4h
21	08:00	2	3	Oral glucose tolerance 0h
22	08:15	2	3	Oral glucose tolerance 15min
23	08:30	2	3	Oral glucose tolerance 30min
24	08:45	2	3	Oral glucose tolerance 45min
25	09:00	2	3	Oral glucose tolerance 60min
26	09:30	2	3	Oral glucose tolerance 90min
27	10:00	2	3	Oral glucose tolerance 120min
28	11:00	2	3	Oral glucose tolerance 3h
29	12:00	2	3	Oral glucose tolerance 4h/Lunch SLD 0h
30	13:00	2	3	Lunch SLD 1h
31	14:00	2	3	Lunch SLD 2h
32	15:00	2	3	Lunch SLD 3h
33	16:00	2	3	Lunch SLD 4h/Physical activity 0h
34	16:15	2	3	Physical activity 15min
35	16:30	2	3	Physical activity 30min
36	16:45	2	3	Physical activity 45min
37	17:00	2	3	Physical activity 60min
38	17:30	2	3	Physical activity 90min
39	18:00	2	3	Physical activity 120min
40	08:00	2	4	Oral lipid tolerance 0h
41	08:30	2	4	Oral lipid tolerance 30min

42	09:00	2	4	Oral lipid tolerance 60min
43	09:30	2	4	Oral lipid tolerance 90min
44	10:00	2	4	Oral lipid tolerance 120min
45	11:00	2	4	Oral lipid tolerance 3h
46	12:00	2	4	Oral lipid tolerance 4h
47	13:00	2	4	Oral lipid tolerance 5h
48	14:00	2	4	Oral lipid tolerance 6h
49	15:00	2	4	Oral lipid tolerance 7h
50	16:00	2	4	Oral lipid tolerance 8h/Cold stress test 0h
51	16:15	2	4	Cold stress test 15min
52	16:30	2	4	Cold stress test 30min
53	16:45	2	4	Cold stress test 45min
54	17:00	2	4	Cold stress test 60min
55	17:30	2	4	Cold stress test 90min
56	18:00	2	4	Cold stress test 120min
